# On the Use of Diversity Measures in Longitudinal Sequencing Studies of Microbial Communities

**DOI:** 10.3389/fmicb.2018.01037

**Published:** 2018-05-22

**Authors:** Brandie D. Wagner, Gary K. Grunwald, Gary O. Zerbe, Susan K. Mikulich-Gilbertson, Charles E. Robertson, Edith T. Zemanick, J. Kirk Harris

**Affiliations:** ^1^Department of Biostatistics and Informatics, Colorado School of Public Health, University of Colorado, Anschutz Medical Campus, Aurora, CO, United States; ^2^Department of Pediatrics, School of Medicine, University of Colorado, Anschutz Medical Campus, Aurora, CO, United States; ^3^Department of Psychiatry, School of Medicine, University of Colorado, Anschutz Medical Campus, Aurora, CO, United States; ^4^Department of Molecular, Cellular and Developmental Biology, School of Medicine, University of Colorado, Anschutz Medical Campus, Aurora, CO, United States

**Keywords:** microbiome, Hill's numbers, repeated measures, alpha diversity, beta diversity, Shannon index, mixed model

## Abstract

Identification of the majority of organisms present in human-associated microbial communities is feasible with the advent of high throughput sequencing technology. As substantial variability in microbiota communities is seen across subjects, the use of longitudinal study designs is important to better understand variation of the microbiome within individual subjects. Complex study designs with longitudinal sample collection require analytic approaches to account for this additional source of variability. A common approach to assessing community changes is to evaluate the change in alpha diversity (the variety and abundance of organisms in a community) over time. However, there are several commonly used alpha diversity measures and the use of different measures can result in different estimates of magnitude of change and different inferences. It has recently been proposed that diversity profile curves are useful for clarifying these differences, and may provide a more complete picture of the community structure. However, it is unclear how to utilize these curves when interest is in evaluating changes in community structure over time. We propose the use of a bi-exponential function in a longitudinal model that accounts for repeated measures on each subject to compare diversity profiles over time. Furthermore, it is possible that no change in alpha diversity (single community/sample) may be observed despite the presence of a highly divergent community composition. Thus, it is also important to use a beta diversity measure (similarity between multiple communities/samples) that captures changes in community composition. Ecological methods developed to evaluate temporal turnover have currently only been applied to investigate changes of a single community over time. We illustrate the extension of this approach to multiple communities of interest (i.e., subjects) by modeling the beta diversity measure over time. With this approach, a rate of change in community composition is estimated. There is a need for the extension and development of analytic methods for longitudinal microbiota studies. In this paper, we discuss different approaches to model alpha and beta diversity indices in longitudinal microbiota studies and provide both a review of current approaches and a proposal for new methods.

## Introduction

Identification of the majority of organisms present in human-associated microbial communities is now feasible with the advent of high throughput sequencing technology. Several studies have shown large subject-to-subject variability (Flores et al., 2014) as well as many different factors that might contribute to variability in microbiome studies, i.e., diet, region, exposure, genetics, etc. Given the highly personalized microbiome, valuable information is likely to come from studies following subjects over time. The use of longitudinal study designs is important to better understand the contribution of the microbiome to human health (Flores et al., 2014). Complex study designs with longitudinal sample collection require analytic approaches to account for this additional source of variability, and to allow examination of changes within subjects.

Extension and development of analytic methods are needed for longitudinal microbiota studies (Gerber, 2014). Current approaches include extending models applied to individual taxa to address repeated measures over time (Chen and Li, 2016; Fang et al., 2016; Wagner et al., 2017) but not much attention has been given to discussion and extension of ecological community indices, which are useful for describing the community biodiversity. The majority of analytic methods for these measures were developed for studying one community over time in the field of ecology. This paper, therefore, focuses on the application and development of methods for diversity indices in order to model multiple communities (i.e., subjects) over time.

Several measures of diversity have been widely applied to microbiota data. The selection of a diversity measure is important as the inferences made can differ widely depending on the measure chosen (Jost, 2006; Ellison, 2010; Tuomisto, 2010a,b; Jurasinski and Koch, 2011; Moreno and Rodriguez, 2011; Tuomisto, 2011) and several analyses include multiple measures which makes consolidating the results challenging. For alpha diversity, the calculation and comparison of diversity curves (Renyi, 1961; Whittaker, 1972; Hill, 1973; Carranza et al., 2007; Studeny et al., 2011; Gotelli and Chao, 2013) has been proposed, which alleviates the need to choose a single diversity index. These curves provide a useful visualization but there currently is no method available to make inferences about the changing shape of the curves over time.

Furthermore, it is possible that no change in alpha diversity (single community/sample) may be observed despite the presence of a highly divergent community composition. Thus, it is also important to use a beta diversity measure (similarity between multiple communities/samples) that captures changes in community composition. Ecological methods developed to evaluate temporal turnover have currently only been applied to investigate changes of a single community over time (Collins et al., 2000; Korhonen et al., 2010; Yuan et al., 2016; Lewthwaite et al., 2017). In order to evaluate changes over time in multiple communities (i.e., subjects), an extension to a hierarchical model is needed.

In this paper, we discuss different approaches to model diversity indices in longitudinal microbiota studies. All approaches are illustrated using a motivating example described in section Description of Motivating Example. In section Single Alpha Diversity Index, a linear mixed model (also called a hierarchical model) is used to separately model three alpha diversity measures over time and the results are compared across measures. The recently proposed alpha diversity curves are explained in section Alpha Diversity Curves and we develop a hierarchical model approach to analyze these curves longitudinally with a non-linear mixed model. In section Beta Diversity, a description of how to model beta diversity in longitudinal studies is provided. This work provides both a review of current approaches and presents newly developed methods.

## Description of motivating example

The motivating example used throughout this paper is a prospective study of 50 subjects aged 10–22 years with cystic fibrosis (CF) and admitted for intravenous (IV) antibiotic therapy for a pulmonary exacerbation (Pex). All subjects were treated following standard clinical guidelines, at the discretion of their physician. Study evaluation and specimen collection occurred at three times, hospital admission (day 0–2; Beg Pex), hospital discharge (day 6–21; End Pex), and a clinical follow-up visit post-exacerbation (within 30 days of completing IV antibiotic treatment; Post Pex). A total of 123 sputum samples were collected and frozen prior to analysis: 31 subjects provided samples at all three times, 12 subjects missed 1 sample collection, and 7 subjects missed 2 sample collections. All models used for the analysis of this dataset assume data are missing at random. Written informed consent was obtained from all patients aged 18 years or older and from parents/legal guardians for patients under 18 years of age, and assent was obtained from patients aged 10–17 years. The study was approved by the Colorado Multiple Institutional Review Board (COMIRB #07-0365).

Bacterial profiles were determined by broad-range amplification and sequence analysis of 16S rDNA following previously described methods and validated in prior publications (Hara et al., 2012; Markle et al., 2013; Zemanick et al., 2017). Quality control procedures were performed on paired-end sequences (Zemanick et al., 2017). Assembled sequences were aligned and classified at the lowest taxonomic level with SINA version 1.2.11 (Pruesse et al., 2012) using the SILVA111 database (Quast et al., 2013) as reference configured to yield the SILVA taxonomy (www.arb-silva.de). Sorted paired-end sequence data were deposited in the National Center for Biotechnology Information Sequence Read Archive (www.ncbi.nlm.nih.gov/sra) under accession number SRP143768. Operational taxonomic units (OTUs) were produced by clustering sequences with identical taxonomic assignments (generally genus level groups). This process generated 20,183,481 sequences for 361 samples (average sequence length: 316 nt; average sample size: 83,722 sequences/sample; minimum sample size: 2,188; maximum sample size: 422,831). The median Goods coverage score was ≥ 99.25% at the rarefaction point of 2,188 (the minimum number of sequences for all samples). The software package Explicet version 2.10.5 (www.explicet.org) (Robertson et al., 2013) was used for calculation of diversity indices at the rarefaction point. Taxonomic data utilized in this analysis have been included as Supplementary Material and represent a subset of data from the parent study (excluding saliva samples and samples from repeated Pex events).

## Single alpha diversity index

Diversity, defined as the description of “the variety and abundance of species in a defined unit of study,” (Magurran, 2004) is a measure often used to describe the complexity of a community. Several measures of diversity have been widely applied to microbiota data and have been used previously as outcomes in longitudinal models (Gajer et al., 2012; Flores et al., 2014; Wagner et al., 2017). In this section we similarly apply linear mixed models to three diversity measures over time. These results serve as a useful comparator for the remaining sections of this paper.

### Differences in weights for evenness and richness components across measures explain differences in results

Diversity indices applied to microbiota data consist of differing weights of two components, richness and evenness (Jost, 2006). Richness is a count of the number of different taxa observed in the community without regard to their frequencies, and evenness refers to the equitability of the taxa frequencies in a community. Three commonly used alpha diversity measures include species observed, Shannon index and Simpson index:

$$\begin{array}{l} S\left(obs\right)=\;\sum_{k}I\left(p_{k}>0\right)\\ Shannon\;=\;-\sum_{k}p_{k}\;ln\left(p_{k}\right)\\ Simpson=\sum_{k}p_{k}^{2}, \end{array}$$

where *p* is some function of frequency, often relative abundance (proportion of total sequences) for each taxon, *k*.

Species observed is equal to richness and therefore provides no weight to the evenness component, Shannon index equally weights richness and evenness and Simpson index provides more weight to evenness (Jost, 2006). Moreover, the units are different across the measures, species observed is a count, Shannon index contains a logarithmic value and Simpson index is a sum of squared proportions. These differences in weighting and units explain differences often observed in results from each measure.

### Motivating example

**Figure 1:**
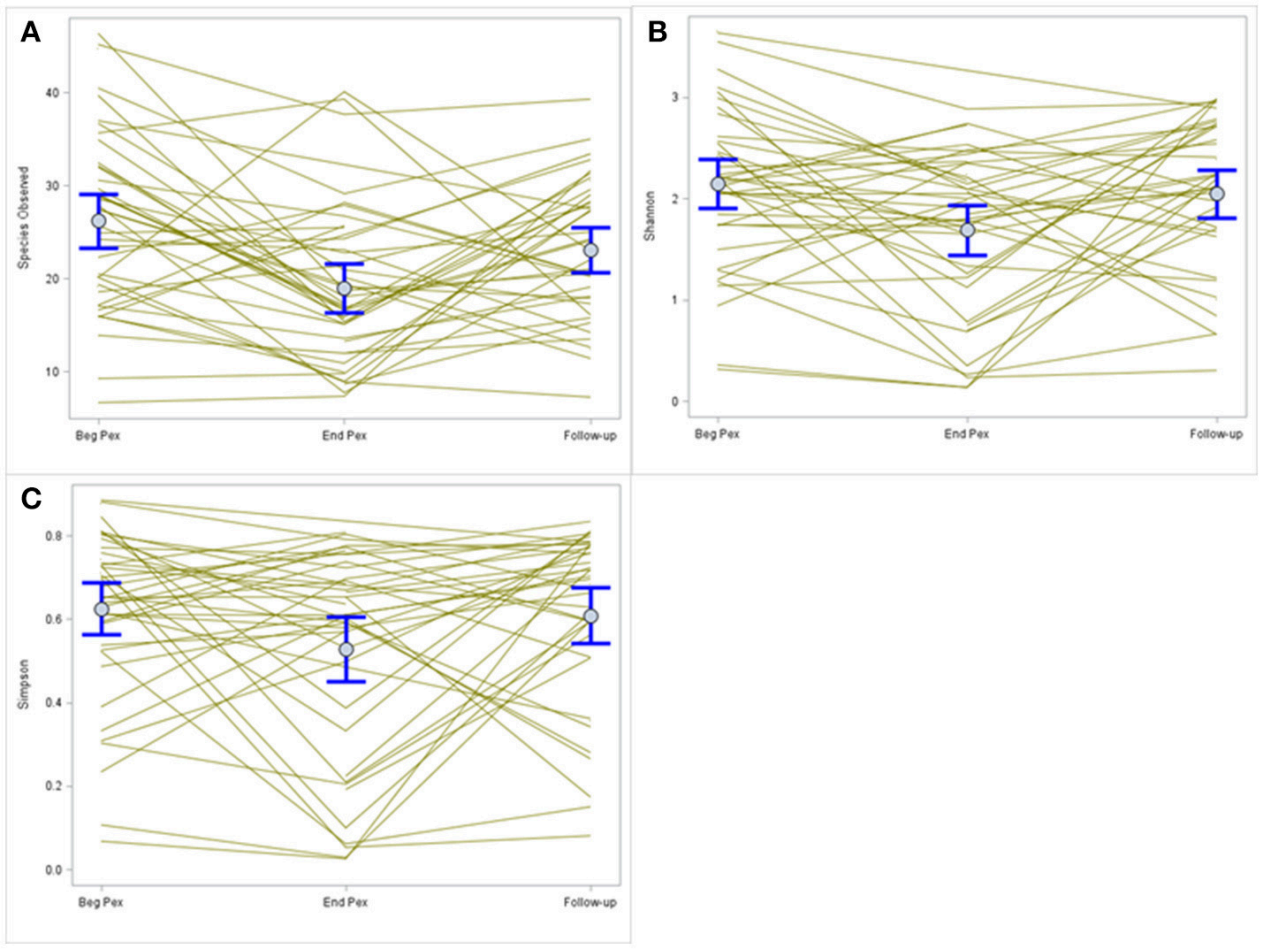
Comparison of alpha diversity over time. Species observed **(A)** shows a decrease in values after completion of IV antibiotic treatments that increase at follow-up. A similar pattern is observed for the Shannon and Simpson diversity indices **(B,C**, respectively) but the magnitude of change differs for each index.

Species observed, Shannon diversity index and Simpson diversity, as well as the corresponding evenness components, were separately modeled over time in CF patients during a Pex using a linear mixed model that included a random subject intercept with SAS PROC MIXED software. All three diversity measures show a decrease at the end of the Pex (hospital discharge), followed by an increase at follow-up, although measures still remained lower at follow-up than at the beginning of the Pex (Figure 1). Despite this agreement in general trends, the pairwise comparisons of times differ across the measures. The means at each time (Table 1) differed significantly across all three times for species observed and Shannon index (*p* < 0.01), but Simpson diversity differed only marginally across times (*p* = 0.07). Neither of the evenness measures change significantly over time.

**Table 1:** Comparison of common alpha diversity measures at three time points: Beg Pex, End Pex, and a follow-up visit post-Pex.

		**Species observed**	**Shannon Evenness**	**Shannon**	**Simpson Evenness**	**Simpson**
Means (SE)	Beg Pex	26.0 (1.3)	0.46 (0.02)	2.13 (0.12)	0.026 (0.002)	0.62 (0.03)
	End Pex	19.0 (1.3)	0.40 (0.02)	1.70 (0.12)	0.029 (0.002)	0.53 (0.03)
	post-Pex	23.1 (1.4)	0.46 (0.02)	2.07 (0.12)	0.028 (0.002)	0.62 (0.03)
*P*-values	Across all times	**<0.01**	0.09	**0.01**	0.40	0.07
	Beg vs End	**<0.01**	0.06	**<0.01**	0.19	**0.04**
	Beg vs post-Pex	0.10	0.89	0.69	0.27	0.90
	End vs post-Pex	**0.02**	0.05	**0.02**	0.69	0.06

*P-values < 0.05 are indicated in bold*.

### Issues of numerous measures

Although the concept of diversity is rather straightforward, its application can be complicated for several reasons: (1) there are numerous commonly used diversity indices which can yield different results; (2) the nomenclature currently in use to describe diversity is complex and confusing; (3) partitioning diversity into components, such as richness and evenness, may be useful, but varies depending on the diversity measure; and (4) the application to sequence data is complicated by incomplete sampling, i.e., not all bacterial sequences may be measured due to differences in sequencing depth. These issues result in debates and general confusion over which diversity measure to use, misinterpretation of results, and an inability to compare results across studies.

Often these indices are incorrectly treated as interchangeable measures of the same characteristic without consideration of the variations in the mathematical properties of each diversity index. The measurement of diversity has been discussed in the ecological literature (Jost, 2006; Ellison, 2010; Jurasinski and Koch, 2011; Moreno and Rodriguez, 2011; Tuomisto, 2011) and there has been an acknowledgement within the field that more rigor is needed. One approach is the calculation and comparison of diversity curves (Renyi, 1961; Whittaker, 1972; Hill, 1973; Carranza et al., 2007; Studeny et al., 2011; Gotelli and Chao, 2013) which provides information across multiple weights of the components of richness and evenness and alleviates the need to choose a single diversity index.

## Alpha diversity curves

The computational formula for diversity curves is

$$\begin{array}{l} D_{\left(q\right)}={\left(\sum_{k=1}^{K}{p_{k}}^{q}\right)}^{\frac{1}{1-q}}, \end{array}$$

where *D* is most commonly calculated for *q* = 0, 1, 2 and *p* is some function of frequency, often relative abundance (proportion of total sequences) for each taxon, *k*, when applied to sequencing data. *D* is undefined for *q* = 1, so the limit as *q* approaches 1 is used instead.

In this equation, the order, *q*, determines how much weight is given to abundant vs. rare taxa (evenness). Species observed (*q* = 0) weights rare taxa more heavily since the abundance of each taxon is not considered. Conversely with diversity of orders > 1 (e.g., Simpson *q* = 2), more weight is given to the more abundant species. Only when *q* = 1 [Shannon index, specifically exp(Shannon index)] are the rare and abundant species equally weighted (Jost, 2006).

A plot of *D* vs. varying values of *q* can provide a more complete way to convey diversity of a community compared to using a single measure (Tothmeresz, 1995; Carranza et al., 2007; Lozupone et al., 2007; Studeny et al., 2011; Gotelli and Chao, 2013; Buckland et al., 2017). For instance, the shape of the curve conveys the evenness of a community. A perfectly even community is represented by a horizontal line (*D* does not change as *q* increases) and a highly uneven community is represented by a curve with an initial steep descent as *q* increases, see https://wagnerbd.shinyapps.io/Frontiers/ (snapshots from the shiny app displayed in Supplementary Figure 1).

### Characterization of diversity curves using bi-exponential function

Although visual inspection of diversity curves may identify potential changes in their shape, it is not clear how to make inferences about whether these differences are meaningful. In this section, we propose a method to characterize a sequence of diversity curves using a bi-exponential function.

The D values, alternatively referred to as Hill's numbers (Hill, 1973), are related to the Renyi entropies(*H*_(*q*)_) (Renyi, 1961) as

$$\begin{array}{l} D_{\left(q\right)}={{\left(\overset{K}{\underset{k=1}{\sum }}{p_{k}}^{q}\right)}^{\frac{1}{1-q}}=e}^{H_{\left(q\right)}} \end{array}$$

where Renyi entropies are $H_{\left(q\right)}=\frac{1}{1-q}\ln\left(\overset{K}{\underset{k=1}{\sum }}{p_{k}}^{q}\right)$

Suppose taxa can be divided roughly into two groups, rare and non-rare, based on abundance *p*, and let *k* = 1, ..*K*_1_ for rare taxa with abundance *p*_1_ and *k* = *K*_1_ + 1, .., *K* for non-rare taxa with abundance *p*_2_. Then

$$\begin{array}{l} D_{\left(q\right)}={\left(\sum_{k=1}^{K}{p_{k}}^{q}\right)}^{\frac{1}{1-q}}\;,\\ \approx \;{\left(K_{1}p_{1}^{q}+K_{2}p_{2}^{q}\right)}^{\frac{1}{1-q}} \end{array}$$

where *K*_1_ + *K*_2_ = *K* and since *e*^ln(*x*)^ = *x*

$$\begin{array}{l} \;\approx \;{\left(K_{1}e^{q*\ln\left(p_{1}\right)}+K_{2}e^{q*\ln\left(p_{2}\right)}\right)}^{\frac{1}{1-q}} \end{array}$$

which is now in the form of a bi-exponential function. We can re-parameterize such that

$$\begin{array}{l} \theta_{1}=-\;\ln\left(p_{1}\right),\\ \theta_{2}=-\;\ln\left(p_{2}\right),\\ \theta_{3}=\frac{K_{1}}{K_{1}+\;K_{2}},and\\ \theta_{4}=K_{1}+\;K_{2}\;then\\ D_{\left(q\right)}={\left(\theta_{3}\theta_{4}e^{q\theta_{1}}+\left(1-\theta_{3}\right)\theta_{4}e^{q\theta_{2}}\right)\;}^{\frac{1}{1-q}} \end{array}$$

where θ_4_ is the total number of taxa in the sample, θ_3_ is the proportion of rare taxa with a fast rate of decline θ_1_ for increasing *q* and θ_2_ is the slow rate of decline in the curve for the 1−θ_3_ proportion of non-rare taxa.

### Development of a hierarchical model

In order to make inferences in the changing shape of the curves over time, we propose a longitudinal model to simultaneously estimate the parameters describing the change in the diversity curves over time. To further simplify the model, we will replace the θ_4_ parameter with the observed number of taxa and drop the 1/(1-q) exponent. Let

$$\begin{array}{l} \theta_{ijm}=\alpha_{jm}+s_{im}\\ D_{\left(q\right)ij}=K_{ij}\theta_{ij3}e^{q\theta_{ij1}}+K_{ij}\left(1-\theta_{ij3}\right)e^{q\theta_{ij2}}+e_{ij} \end{array}$$

where *m* = {1, 2, 3} indexes the θ parameters for the bi-exponential, *i* = 1, .., *n* indexes subjects, *j* = 1, 2, 3 indexes time, α_*jm*_ is the estimated mean for parameter *m* at time *j*, $e_{ij}\;\sim \;N\left(0,\sigma^{2}\right)$ is a random error, and *s*_*im*_ is a random subject intercept,

$$\begin{array}{l} s_{im}\;\sim \;N\left(\left[\;\begin{array}{c} 0\\ 0\\ 0 \end{array}\right]\;,\left[\begin{array}{ccc} d_{11} & d_{12} & d_{13}\\ d_{21} & d_{22} & d_{23}\\ d_{31} & d_{32} & d_{33} \end{array}\right]\;\right) \end{array}$$

### Motivating example for alpha diversity curves

**Figure 2:**
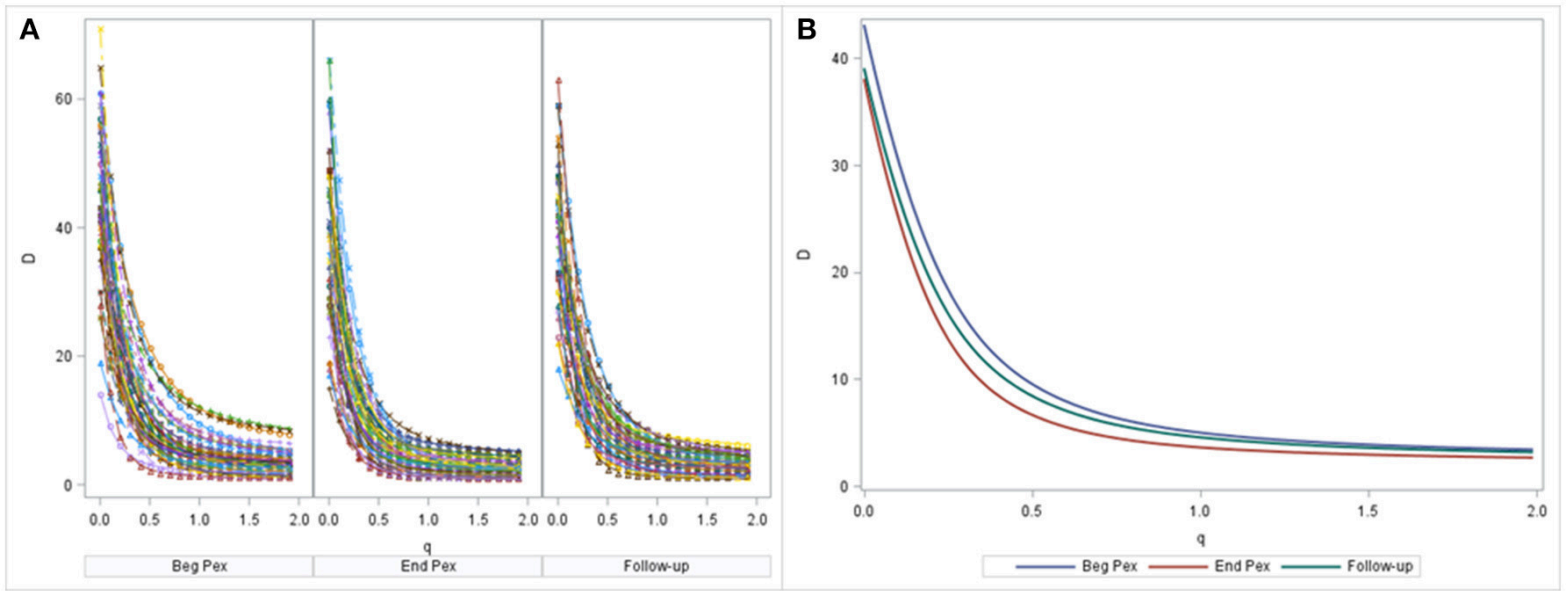
Diversity curves from each sample, where the points correspond to D values from the Hill's numbers (y-axis) plotted vs. the *q* values (x-axis). The corresponding bi-exponential distribution fits are displayed using lines for each time point separately **(A)**. The average diversity curves at each time estimated from the joint longitudinal model are displayed in panel **(B)**.

A non-linear mixed model was estimated by maximum likelihood using SAS PROC NLMIXED. Diversity curves were calculated for each sample and are presented graphically for each subject at each time (Figure 2A). The fitted curves follow points indicating good fit. All curves have similar shape and show curvature indicating that the bacterial communities of all samples are relatively uneven (a flatter curve would indicate a more even community). The curves appear to exhibit steeper decline from beginning of the Pex (Beg Pex) to the follow-up visit (post Pex) for the majority of subjects.

The mean curves at each time from the longitudinal model indicate that *D*(0) (species observed, i.e., richness) was highest at the beginning of the pulmonary exacerbation and decreased thereafter (Figure 2B). The curve for the end of the pulmonary exacerbation (End Pex) is more uneven (steeper decline) compared to the other two times. The parameter estimates provided in Table 2 correspond to visual observations related to the change in shape in both the individual curves and the mean curves, but in addition provide quantification and the ability to make inference on the change in the shape of the curves over time. Estimated θ_1_ at End Pex is largest, corresponding to the visually steepest decline, θ_2_ estimates increase over time resulting in lower diversity at the Post Pex time associated with the more dominant taxa, and θ_3_ estimates from the hierarchical model indicate a significant shift toward a lower proportion of rare taxa over time (Table 2).

**Table 2:** Parameter estimates from nonlinear mixed model at three time points: Beg Pex, End Pex, and a follow-up visit post-Pex.

**Est (95% CI)**	**Beg Pex**	**End Pex**	**post-Pex**
θ_1_	3.65 (2.97–4.33)	3.87 (3.19–4.55)	3.70 (3.02–4.38)
θ_2_	1.48 (0.90–2.06)	1.63 (1.05–2.21)	1.64 (1.06–2.22)
θ_3_	0.82 (0.72–0.91)	0.79 (0.69–0.90)	0.62 (0.47–0.77)

The shapes of the diversity curves differ (Figure 2B) which explains the discrepancies in comparing the diversity indices that were observed earlier (Table 1). In addition, using the individual indices conveys no information about evenness without calculating an evenness measure separately. Although separate models evaluating changes in diversity, evenness and richness are easily obtained, there is a different evenness measure corresponding to each diversity measure and therefore this approach suffers from the same issue of multiple measures that may provide different answers depending on how much weight is given to rare taxa. Advantageously, diversity curves provide information about the change in the evenness of the communities over time in a single model. These characteristics can be evaluated and compared numerically with the longitudinal model that allows estimation of trends in the four parameters from the bi-exponential distribution and additional estimates of non-linear functions of these parameters. Application of the hierarchical model to the parameters from the bi-exponential distribution represents a novel approach to evaluating changes in the diversity curves over time.

## Beta diversity

In addition to partitioning diversity into independent components describing evenness and richness, we can also partition diversity by collections of samples. Whereas, the diversity associated with a single sample is referred to as a local (alpha) component, the diversity of the collection of samples is referred to as the regional (gamma) component and the relationship between these two is referred to as beta diversity (Legendre and Legendre, 1998). Previously, an alpha diversity measure was calculated for each sample α(*x*_*ij*_), here, a beta diversity index is calculated for each pair of samples $\beta \left(x_{ij},x_{ij^{\prime }}\right)$, and represents either a similarity or a distance between the two samples.

Changes in alpha diversity over time can be useful for evaluating the change in the community structure over time as previously discussed. However, these measures do not convey any information about changes in the community composition (Yuan et al., 2016; Buckland et al., 2017), for example, a community can experience a complete shift in composition, where no taxa are shared, but can still have similar alpha diversity measures, i.e., similar number and abundance of taxa. An important addition to evaluating a microbial community over time in any longitudinal analysis is the incorporation of beta diversity.

As with the alpha diversity measures, there are several possible beta diversity indices that one could use, some of the most popular in microbiome studies include Jaccard, Bray-Curtis, Morisita-Horn and Sorenson. Similar to the earlier discussion of alpha diversity measures, differing results are obtained across beta diversity indices, again due to differences in weighting of the components (Tuomisto, 2010a,b). The calculation of beta diversity indices for all combinations of pairs of samples results in a distance matrix that is often used for ordination (e.g., principal coordinates analysis) and data exploration in microbiota data analysis. Several methods are available for analysis of the full distance matrix (correspondence analysis, redundancy analysis, Mantel test, etc.) (Tuomisto and Ruokolainen, 2006). We focus here on regression based methods that allow for inference at the subject level in a longitudinal design, i.e., studying changes over time within a subject. The implication of this focus is that not all values in the distance matrix are of interest, only those that are comparisons of samples collected within a subject.

### Pairwise comparison of consecutively collected samples

In order to evaluate beta diversity indices at the subject level and compare values over time or across groups, specific values from the full distance or similarity matrix are selected for analysis. In the case of longitudinal studies, we are most interested in evaluating changes in the community over time within a subject and can therefore select the distance measures between samples collected on the same subject $\beta \left(x_{ij},x_{ij^{\prime }}\right)$. One approach that has been used is to calculate the mean or median beta diversity value for each subject and use this as an outcome (Gajer et al., 2012). Here we instead use the beta diversity values from consecutively collected samples within the same subject β(*x*_*ij*_, *x*_*ij*+1_) as outcomes in a second stage generalized linear mixed model.

### Community turnover

A recently proposed approach in the ecological literature is to use beta diversity indices to evaluate temporal turnover (Collins et al., 2000; Shimadzu et al., 2015). Here, the beta diversity indices are regressed on a time lag variable using a time series model. With this approach, all pairwise indices comparing samples within a subject are used $\beta \left(x_{ij},x_{ij^{\prime }}\right)$ (not simply the indices from consecutive samples as above) and a rate of change in composition is estimated. The proposed approach has been useful for assessing turnover in a single community over time (Collins et al., 2000; Korhonen et al., 2010; Yuan et al., 2016; Lewthwaite et al., 2017), but requires extension to a hierarchical model to make inferences on groups of communities (i.e., subjects in our motivating example). We suggest the use of a similar model to that used for the indices of consecutive samples and simply replace the single independent time variable with one denoting all pairs (Wagner et al., 2017).

### Shannon beta

Another useful measure that has been proposed in the ecological literature (Marcon et al., 2012, 2014) and applied to microbiota data (Zemanick et al., 2015) is the Shannon Beta index. This measure can be decomposed into multiple alpha and beta components even when community weights are unequal (Tuomisto, 2010a,b; Marcon et al., 2012). Thus, in addition to being widely used in other disciplines, its well-understood mathematical properties and underlying theory make Shannon Beta a useful measure overall.

This approach extends the beta diversity measure to apply to a collection of samples rather than just for pair-wise comparisons $\beta \left(x_{ij},x_{ij^{\prime }},x_{ij^{\prime \prime }},..\right)$. For our example, Shannon Beta (*H*_β_*i*__) is calculated as

$$\begin{array}{l} H_{\beta_{i}}=\underset{j}{\sum }\frac{c_{ij}}{c_{i++}}\underset{k}{\sum }\frac{c_{ijk}}{c_{ij+}}\;ln\;\left(\frac{\frac{c_{ijk}}{c_{ij+}}}{\frac{c_{i+k}}{c_{+++}}}\right) \end{array}$$

where *c*_*ijk*_ is the sequence count for subject *i*, from time *j* and taxon *k*, the + in the subscript denotes the summation of the counts over the specified indicator.

For ease of clinical interpretation, Shannon Beta is expressed as a Hill's number which indicates the effective number of communities represented by the collection of samples or the number of distinct communities. This measure is dependent on the number of samples from which it was calculated, and ranges from 1 to 3 in our motivating example. A normalizing transformation was used to rescale the Hill's numbers to allow comparison across subjects with differences in the number of collected samples (Chao et al., 2010).

$$\begin{array}{l} H_{ni}=\frac{H_{\beta_{i}}-1}{j_{i}-1} \end{array}$$

where *j*_*i*_ is the number of samples for subject *i*.

### Motivating example for beta diversity

Morisita-Horn (MH, Beta-diversity) values for pairwise samples *j* and *j*′ within each subject *i* were calculated as follows

$$\begin{array}{l} Morisita\;Horn\left(x_{ij},x_{ij^{\prime }}\right)=2\left(\frac{\sum_{k}\;\left(c_{ijk}*\;c_{ij^{\prime }k}\right)}{\left(\frac{\sum_{k}{c_{ijk}}^{2}\;}{{c_{ij+}}^{2}}+\frac{\sum_{k}{c_{ij^{\prime }k}}^{2}\;}{{c_{ij^{\prime }+}}^{2}}\right)\left(c_{ij^{\prime }+}*c_{ij^{\prime }+}\right)}\right) \end{array}$$

**Figure 3:**
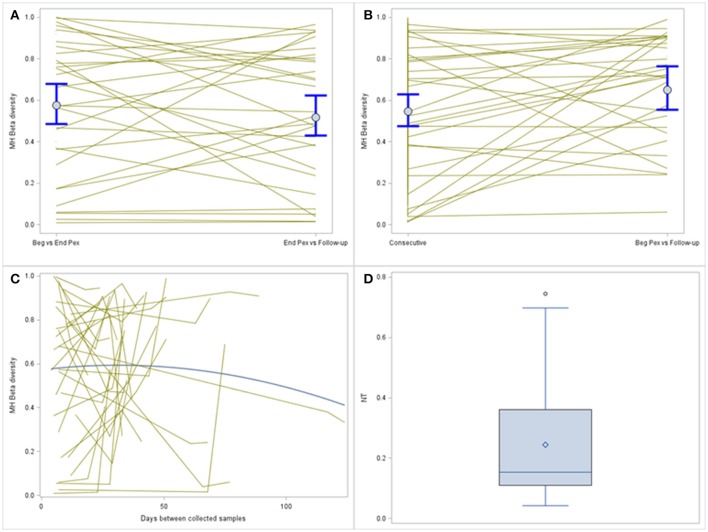
Comparison of MH beta diversity measures for the consecutively collected samples **(A)** and plotted vs. time lag **(B)**. Each subjects value is plotted and connected with lines and the means and 95% confidence intervals from the generalized linear models are plotted with dots and whiskers. The bottom panel displays the MH beta diversity measures plotted over actual time between sample collection **(C)**, individual subjects are indicated by the thin gray lines and the thicker blue line indicates the average change. The distribution of the normalized Shannon Beta diversity measures for all subjects **(D)**.

MH was compared over time using a log-normal model and included a random subject effect. MH in this example is a similarity measure bound between 0 and 1. Values closer to 1 indicate the pair of samples are more similar. MH values, on average, are similar for the two consecutive sample pairs (Beg vs. End Pex and End Pex vs. Follow-up), but individual subjects have varying patterns (Figure 3A). Specifically, there are several subjects with limited similarity between communities (MH for both consecutive pairs is close to 0). The turnover analysis was performed in two ways, first, time was defined using the clinically meaningful states (Beg Pex, End Pex, and Post Pex) and second, time was defined using the number of days between when samples were collected. The latter approach is used for illustrative purposes to better show the differences between the consecutive and turnover analyses for this particular example given the small number of samples collected per subject. The turnover analysis using the clinically meaningful time points (Figure 3B) reveals that the bacterial communities at Post Pex are more similar to the communities at the Beg Pex than the other comparisons (in this case the consecutive sample comparisons: Beg vs. End Pex and End Pex vs. Post Pex). This indicates that the communities are converging back to the original communities observed at the beginning of the Pex after being perturbed by antibiotics. This is also evident in those subjects with very different communities between consecutively collected samples but show a much higher degree of similarity between Beg Pex and Post Pex. This same pattern is seen using the continuous version of the time variable, where the average similarity values increase with increasing time lag between pairs up to approximately 45 days, after which the similarity declines over time (Figure 3C). These figures also illustrate the large amount of variability across subjects with varying patterns in change over time. For both turnover analyses, there are individual subjects whose communities remain stable (no change in similarity with increasing time lag) and those whose communities indicate a directional change (similarity decreases with increasing time lag). A hierarchical model allows each subject's trajectory to deviate from the overall average, capturing this between subject variability. It may be useful to further evaluate the estimated individual subject trajectories by identifying subjects with specific patterns of change over time or by identifying groups of subjects with similar trajectories.

A single Beta diversity measure, Shannon Beta diversity, was calculated for each subject to quantify the number of bacterial communities represented. The median of the beta diversity values after normalization was 0.15 and ranged from 0.04 to 0.75 (Figure 3D). Higher values indicate that more distinct communities were observed for a subject, this value ranges from 0 to 3 (number of samples collected per subject). For the subset of subjects with all three samples collected, the median of the Hill's beta diversity measure was 1.3 and ranged from 1.1 to 2.2 and 50% of the values were between 1.2 and 1.6 indicating that the majority of subjects did not experience large shifts in their bacterial communities across all three time points as the number of distinct communities (i.e., Hill's numbers) were around 1.

**Figure 4:**
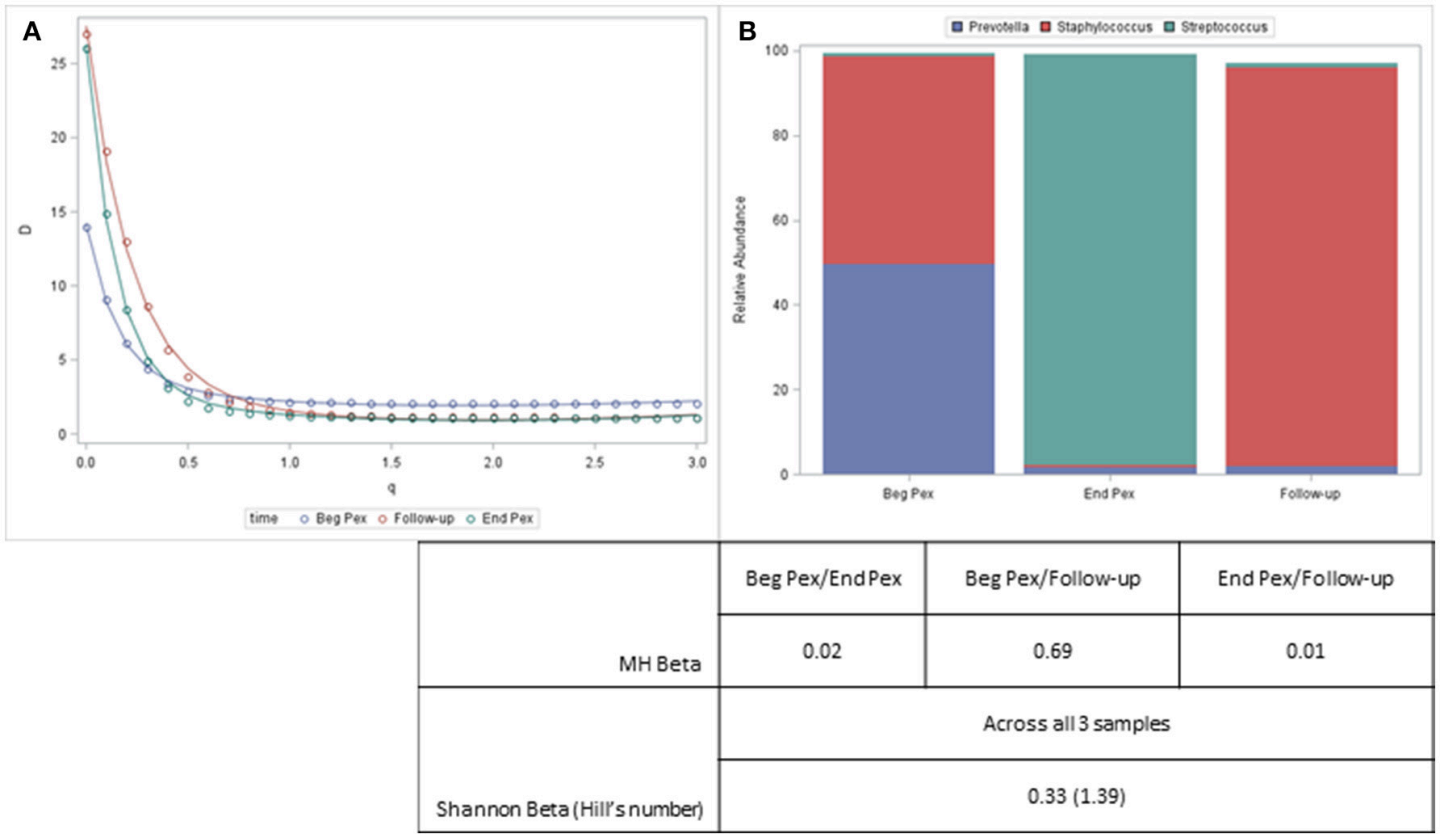
Diversity curves for an example subject **(A)** corresponding to the communities represented by the stacked barcharts **(B)**. Taxa with a relative abundance > 5% for any sample are displayed. The table shows the pairwise MH beta diversity values and the Shannon Beta for this subject.

Both alpha and beta diversity measures from a single example subject are displayed in Figure 4. For this subject, the Shannon diversity (*q* = 1) decreases for the second time point and then increases at the third time point but remains below the values observed at the first sample. The communities are very uneven (include several rare taxa) and the diversity curves cross each other indicating that different measures would yield different results, especially for the second and third samples which would differ with lower *q* values but show similar community characteristics for larger values. The bar charts display the composition of the three communities and show that despite the second and third sample having similar alpha diversity values, the communities are very different. This information, however, is captured with the beta diversity measures. The pairwise MH similarity values illustrate that the samples collected consecutively differ, but that the first sample and the third sample have similar compositions. This indicates that after antibiotics this subject's bacterial community more closely resembled their starting community. The Hill's number for the Shannon beta measure indicates that approximately 1.4 distinct communities are observed for this subject.

The three different approaches to evaluating beta diversity measures in longitudinal studies discussed here provide additional information about changes in communities over time that are not captured by simply modeling alpha diversity over time. The pairwise measures are useful for identifying subjects or times at which shifts in the community are observed and the turnover analysis can yield insights into whether there are consistent shifts with increasing time between sample collections. The single measure (Shannon beta) calculated for each subject can aid in identifying subjects with similar communities across all the time points or those with large changes that suggest shifts over time.

## Discussion

In this work, a discussion of methods for evaluating diversity measures in longitudinal microbial data includes the commonly used approach of modeling a single alpha diversity measure over time. Modeling one alpha diversity measure over time (e.g., species observed) could result in different inferences than modeling a different alpha diversity measure (e.g., Simpson). Diversity curves and their calculation were reviewed as a way to alleviate the need to select a single measure; however, until now, there has been no discussion of how to compare curves over time quantitatively. We developed an approach that utilizes a bi-exponential distribution to summarize each curve and compare curves over time using a hierarchical model. This represents a contribution to the field of microbiome data analysis. Lastly, we discuss the additional information that is gained by evaluating beta diversity measures to assess changes in community composition over time and implement three different approaches and discuss their differences.

Several measures of diversity have been widely applied to microbiota data and have been used previously as outcomes in longitudinal models (Gajer et al., 2012; Flores et al., 2014; Wagner et al., 2017). Often these indices are incorrectly treated as interchangeable measures of the same characteristic, which has caused debates and general confusion over which diversity measure to use, misinterpretation of results, and an inability to compare results across studies. Diversity curves incorporating aspects of these different measures were promoted as a solution, but until now have been used simply for visualization purposes (Renyi, 1961; Whittaker, 1972; Hill, 1973; Carranza et al., 2007; Studeny et al., 2011; Gotelli and Chao, 2013). We chose to model the diversity curves proposed by Jost (2006) that have been shown to equal several commonly used measures, although we recognize that there are alternative complexity curves that have been proposed (Rajaram and Castellani, 2016). The use of any curve will require a model to be applied to capture the shape of the curve to make inferences about changes over time.

An important addition to evaluating a microbial community over time in any longitudinal analysis is the incorporation of beta diversity, as these measures convey information about changes in community composition. The majority of previous analyses have concentrated on modeling turnover in a single community (Collins et al., 2000; Korhonen et al., 2010; Yuan et al., 2016; Lewthwaite et al., 2017). Two studies (Gajer et al., 2012; Wagner et al., 2017) modeled beta diversity measures over time using a hierarchical model similar to the model using beta diversity from consecutive times discussed in this paper, but the descriptions of the models were relegated to supplements. In this paper we describe in detail the modeling approach and its interpretation. Our example included the Morisita-Horn beta diversity measure, selected because it is not influenced by richness and sequencing effort (Magurran, 2004). However, various other beta diversity measures including phylogenetic measures that account for genetic similarity between taxa can be used (Gotelli and Chao, 2013) without loss of generality of the modeling approach.

The Shannon Beta index is another useful measure that has been proposed in the ecological literature (Marcon et al., 2012, 2014) and applied to microbiota data (Zemanick et al., 2015). This measure provides a single number denoting the similarity across multiple communities and can be used to identify subjects with small or large changes in their bacterial community. To our knowledge, the Shannon Beta index has not been previously applied to evaluate changes in bacterial communities within a subject over time and thus our methods represent a novel application of this measure to longitudinal microbiota data.

All of the methods discussed are illustrated and compared using a motivating example in cystic fibrosis. The example included a small number of repeated samples per subject and samples corresponded to clinically meaningful time points (hospital admission, hospital discharge, and a follow-up visit post-exacerbation). For this reason, the models we employed designated time as a categorical variable. These models are flexible and could include time as a continuous variable instead for studies with more longitudinal samples collected. The separate models for alpha diversity indices indicated that diversity decreased with administration of antibiotics mainly driven by a decrease in richness. This pattern was also observed in modeling the alpha diversity curves and provided the ability to make inferences about the components of diversity (richness and evenness) without requiring a separate model for each measure. Alpha diversity can provide information about changes in community structure but does not provide any information about changes in community composition, to address this, beta diversity measures are needed. The majority of studies utilizing beta diversity, use the measures to perform exploratory data analysis with ordination plots. Here, we chose to focus on models of beta diversity that can be used to test hypotheses about change in community composition over time. We illustrated three different approaches for modeling beta diversity. The first used the beta diversity measure from consecutively collected samples, and showed that the average MH was fairly large indicating similar communities. However, there were subjects with a high degree of dissimilarity between consecutive samples (MH values close to 0), whereas the turnover analysis revealed that for these subjects, there were large changes while on treatment but the follow-up community reverted back to the baseline community after being perturbed with antibiotics. Given the small number of samples collected per person in the motivating example, this pattern could have been discerned by evaluating beta diversity for all three combinations of sample pairs, an example with more samples per person or unbalanced collection (samples collected at different times) might have greater benefit from the insight gained from both analyses. The third approach provided a single measure per subject that compares composition of all three samples. This method does not provide information about trends over time but it can be used to rank subjects based on whether they had large changes or whether the three communities were relatively similar. This information could be useful for correlating with clinically important factors, like whether the subject exhibited clinical improvement with treatment.

It was necessary to select specific approaches/indices to include in this work. We recognize though that different alternatives could have been chosen. Instead of providing an exhaustive list and comparison of all methods, we chose approaches that provided good examples of the concepts with the understanding that the methods discussed generalize to other measures; any beta diversity and any measure could be used as the outcome in the models discussed. Future work could incorporate the efforts to classify beta diversity measures based on differences in weighting of the components (Tuomisto, 2010a,b) for application to longitudinal studies.

In summary, several approaches to analyzing diversity measures in a longitudinal study were discussed and compared, including a novel approach modeling longitudinal patterns in alpha diversity curves over time. Given the importance of repeated sampling of microbial communities, especially in human studies, extension of methods appropriate for longitudinal study designs are needed.

## Author contributions

BW and JH: Conception and design. JH, CR, and EZ: Acquisition of the data. BW and CR: Performed the analysis. BW, JH and GG: Drafting the manuscript for important intellectual content. BW, JH, GG, GZ, SM-G, CR and EZ: Review and revision of manuscript.

### Conflict of interest statement

The authors declare that the research was conducted in the absence of any commercial or financial relationships that could be construed as a potential conflict of interest.
