# Supplementary figures and images for: On the Use of Diversity Measures in Longitudinal Sequencing Studies of Microbial Communities
Source: Front Microbiol. 2018 May 22;9:1037. doi: 10.3389/fmicb.2018.01037 (PMC5972327; doi:10.3389/fmicb.2018.01037)

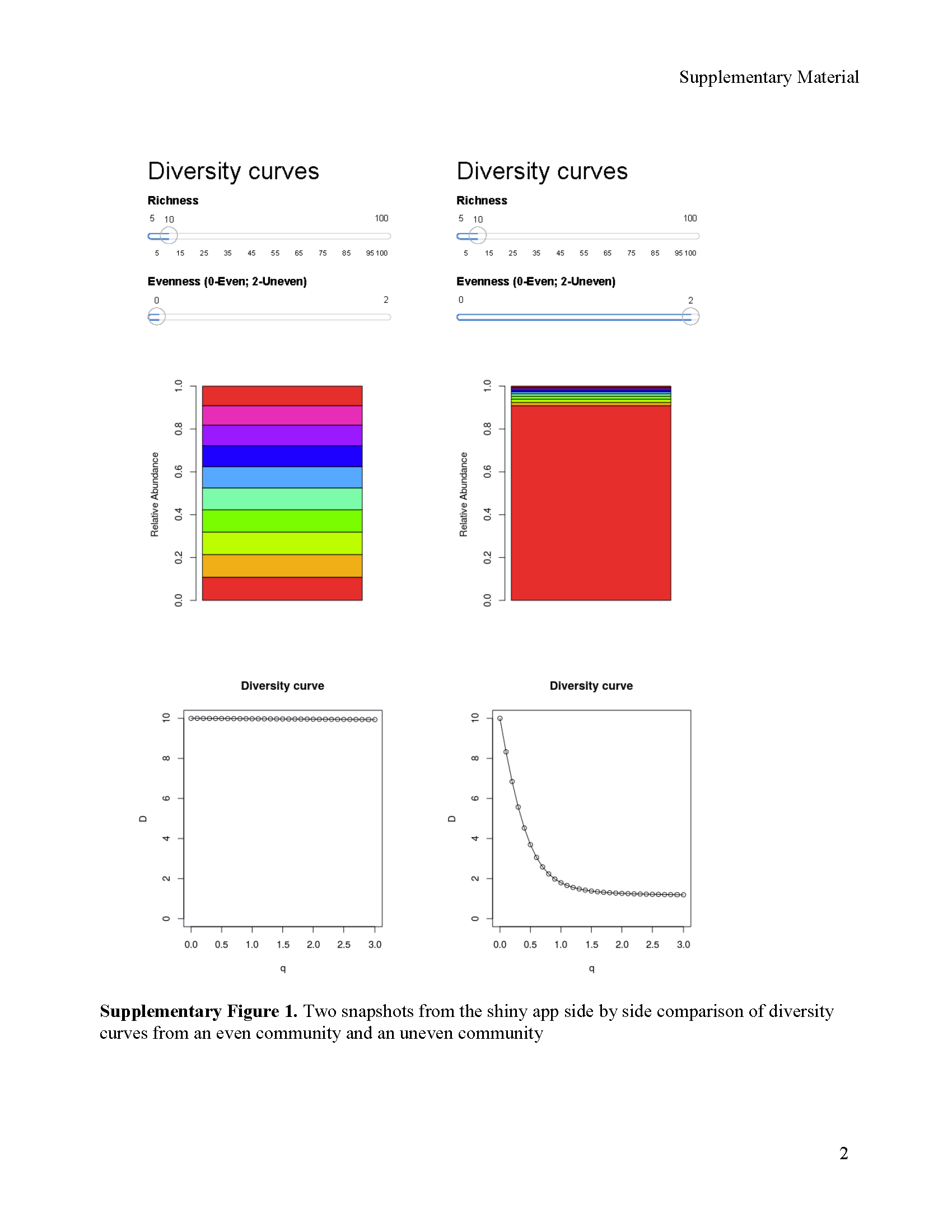

Supplement: Supplementary file 3 [file Image_1.TIFF]
